# 917. Beyond the Burn: Longitudinal Mental Health Impacts in Paediatric Burns

**DOI:** 10.1093/jbcr/irag033.442

**Published:** 2026-04-06

**Authors:** Sudhanvan Iyer, Joshua Wang, Philong Nguyen, Aidan R Carroll, Bovey Liu, Yousef Tanas, George Golovko, Amina El Ayadi, Steven E Wolf, Juquan Song

**Affiliations:** The University of Texas Medical Branch, Pearland, TX; University of Texas Medical Branch, Galveston, TX; University of Texas Medical Branch, Galveston, TX; University of Texas Medical Branch, Galveston, TX; Texas Tech University Health Science Center, Lubbock, TX; Houston Methodist Hospital, Houston, TX; University of Texas Medical Branch, Galveston, TX; Division of Burn and Trauma Surgery, Department of Surgery, University of Texas Medical Branch, Galveston, Galveston, TX

## Abstract

**Introduction:**

Paediatric burns are common traumatic injuries experienced during childhood. In addition to the immediate medical challenges of wound healing, these children are at risk for long-term psychosocial consequences associated with pain, prolonged hospitalization, and social stigmatization. While the psychological aftermath of burns has been extensively studied in adults, the long-term mental health impacts on large paediatric populations remain less defined. This study evaluates the association between paediatric burns and development of psychiatric and behavioral disorders across multiple time intervals.

**Methods:**

Using the TriNetX research network, we conducted a retrospective cohort study of paediatric patients (< 18 years) with burns and compared them to a matched control group without a burn history. Propensity score matching was performed on 23 variables, including demographics, chronic conditions, behavioural diagnoses, and body mass index (BMI). Deceased patients and those with prior psychiatric diagnoses were excluded from both cohorts. Incidence of post-traumatic stress disorder (PTSD), depression, adjustment disorders, and substance use disorders outcomes were analyzed 1, 3, 5, and 5+ years after burn injury. Risk ratios (RRs) with 95% confidence intervals (CIs) were determined.

**Results:**

Matched cohorts contained 25 606 patients with a median follow-up time of 765 days (IQR:1739) for the burn cohort and 1152 days (IQR:1388) for controls. Pediatric burn injuries were associated with persistently higher rates of PTSD, depression, and adjustment disorders though depression significantly wanes between one and three years after injury. Anxiety is initially significantly increased but then returns to control levels at 3 years; substance use disorders do not differ from controls (Table 1).

**Conclusions:**

Paediatric burns are associated with an increased prevalence of psychiatric disorders including PTSD, depression, anxiety, and adjustment disorders compared to propensity matched non-burn controls. Notably, these risks are present 1 year after the initial injury and PTSD, depression, and adjustment disorders are sustained. Anxiety wanes over time and substance use disorders are not different.

**Applicability of Research to Practice:**

Diagnosed psychiatric disorders are higher in burned children which does not appreciably change for PTSD and adjustment disorders. Others improve with time. Future studies should explore the role of age of injury and the effect of directed treatments.

**Funding for the study:**

N/A.

